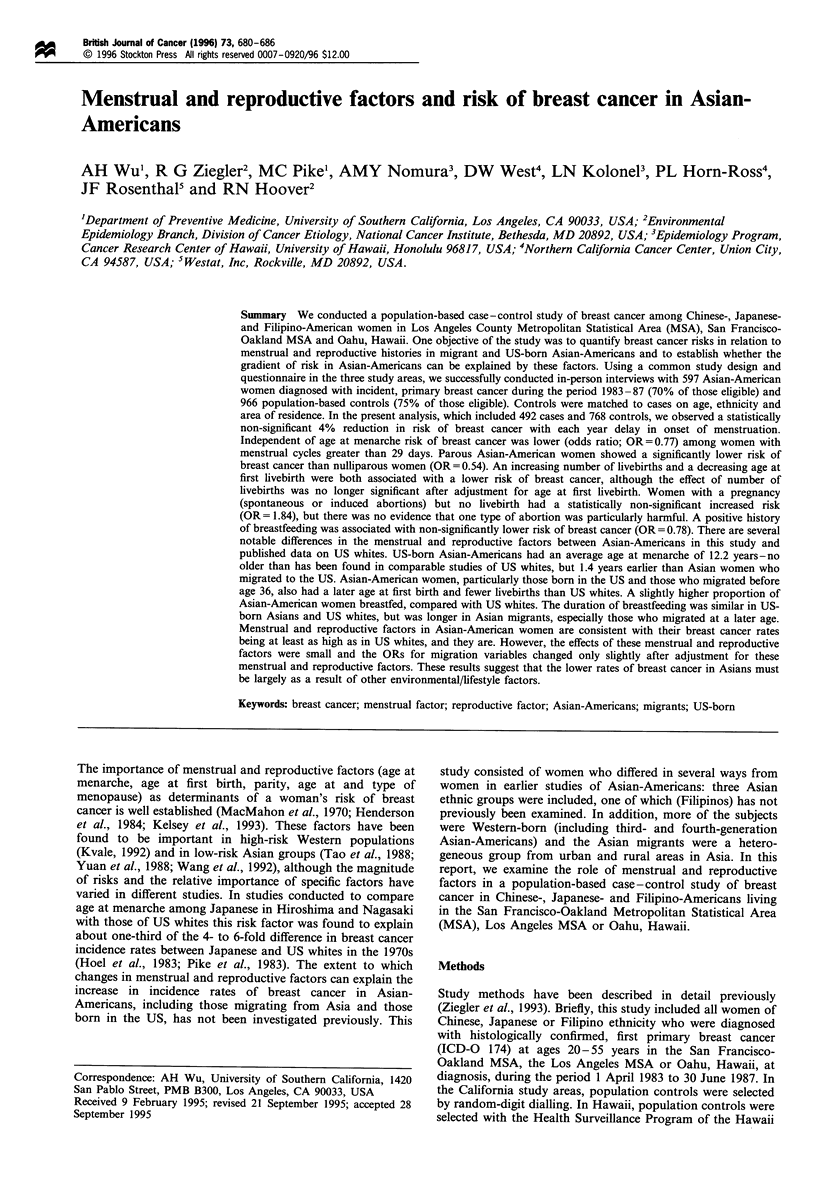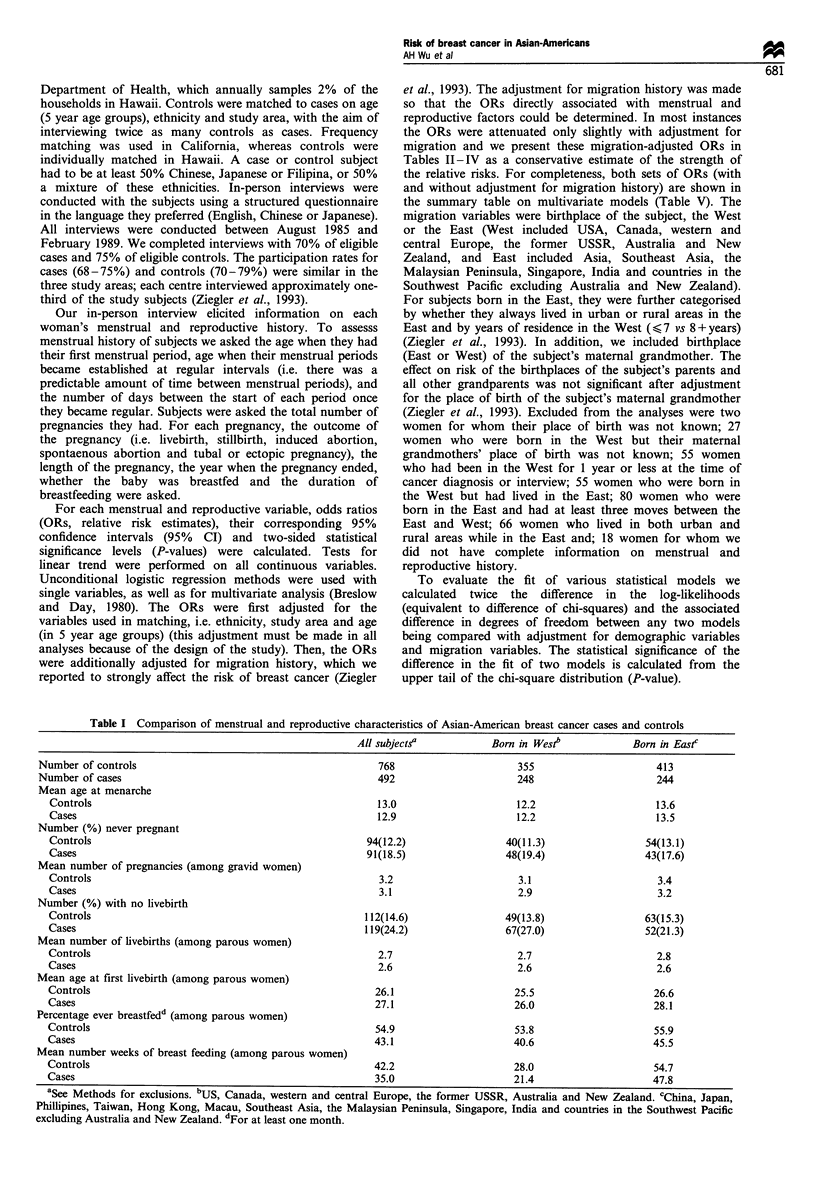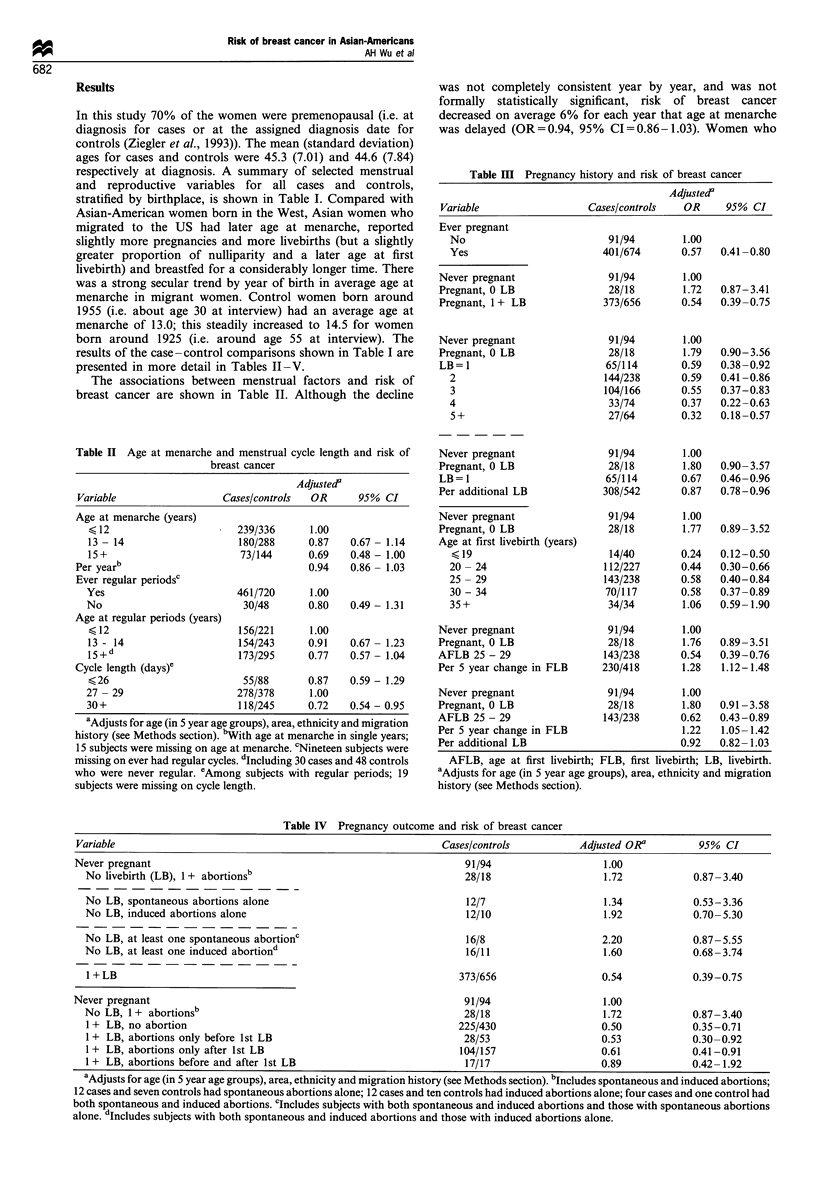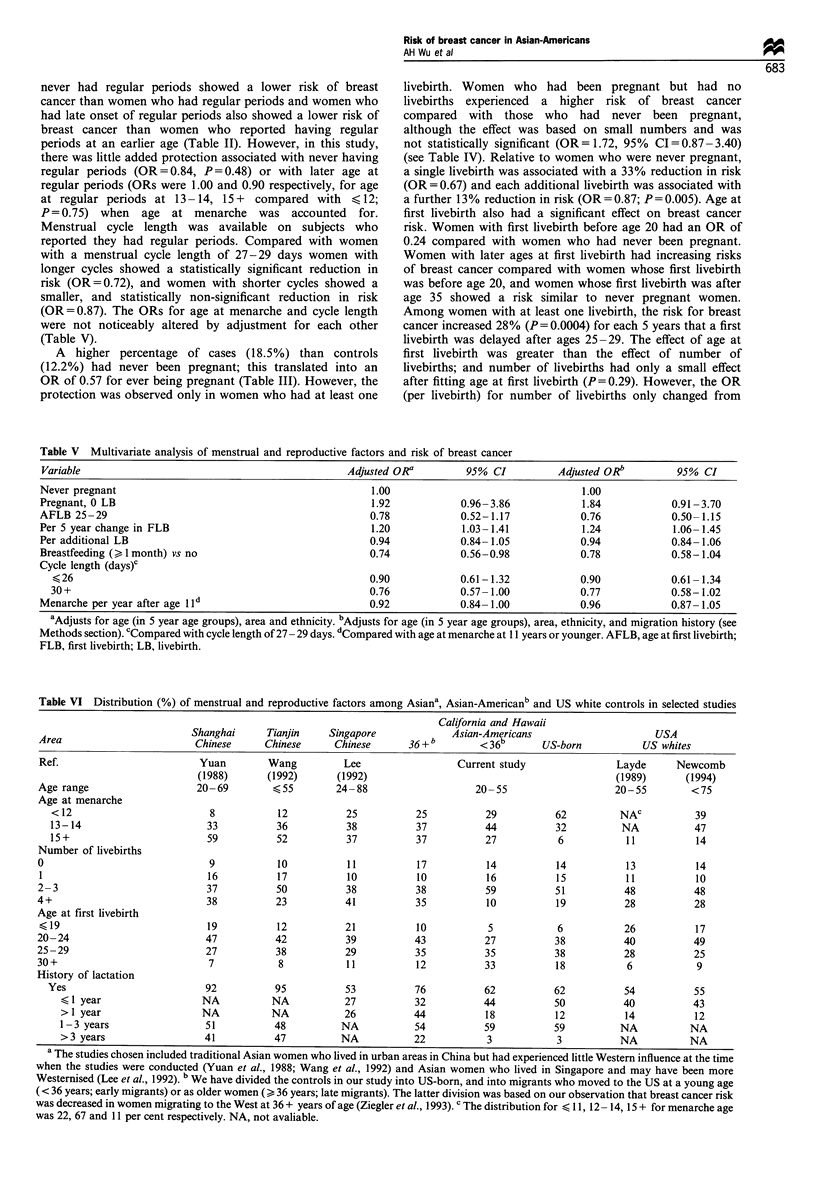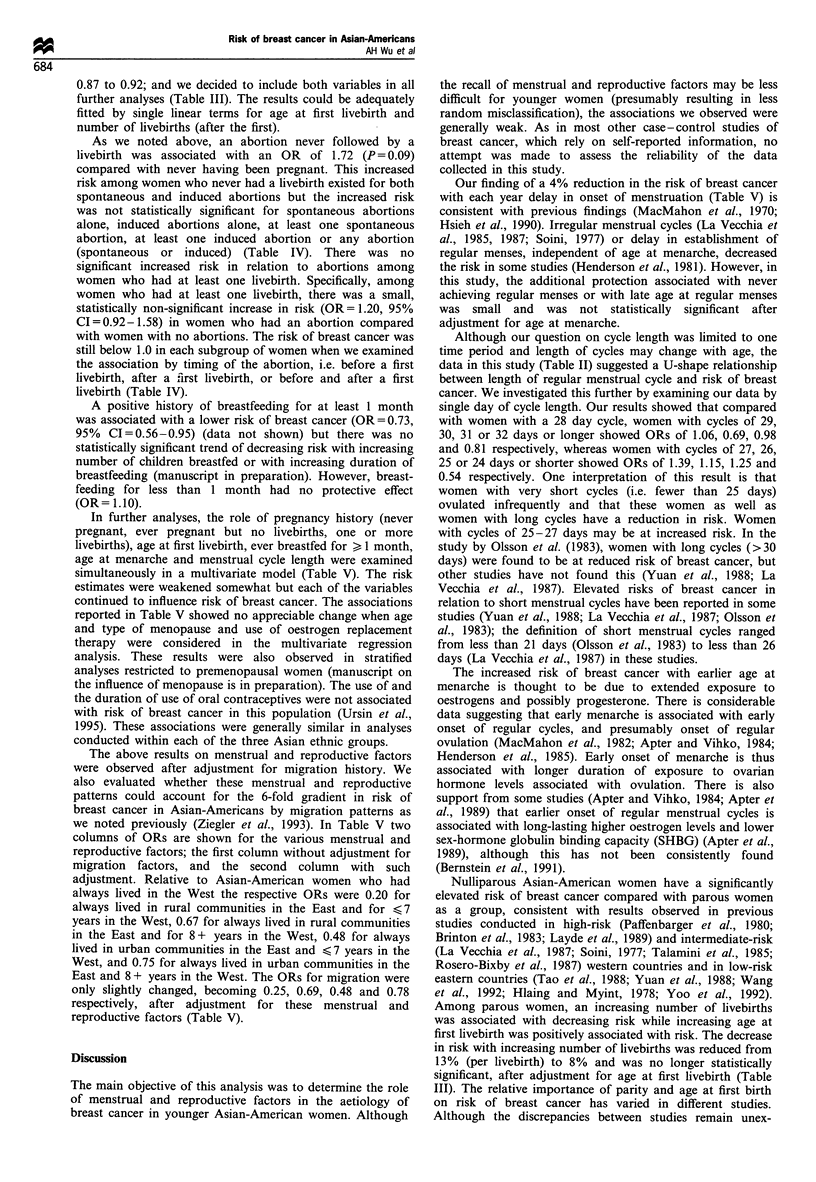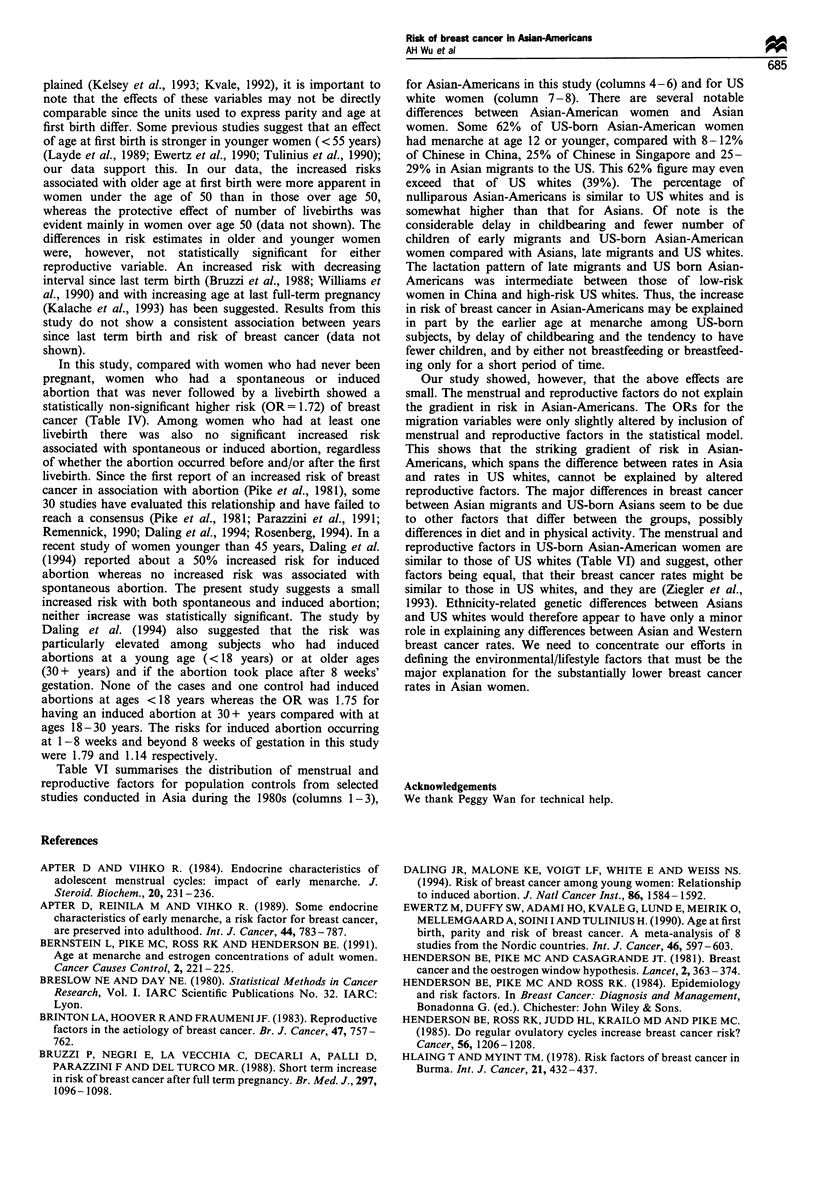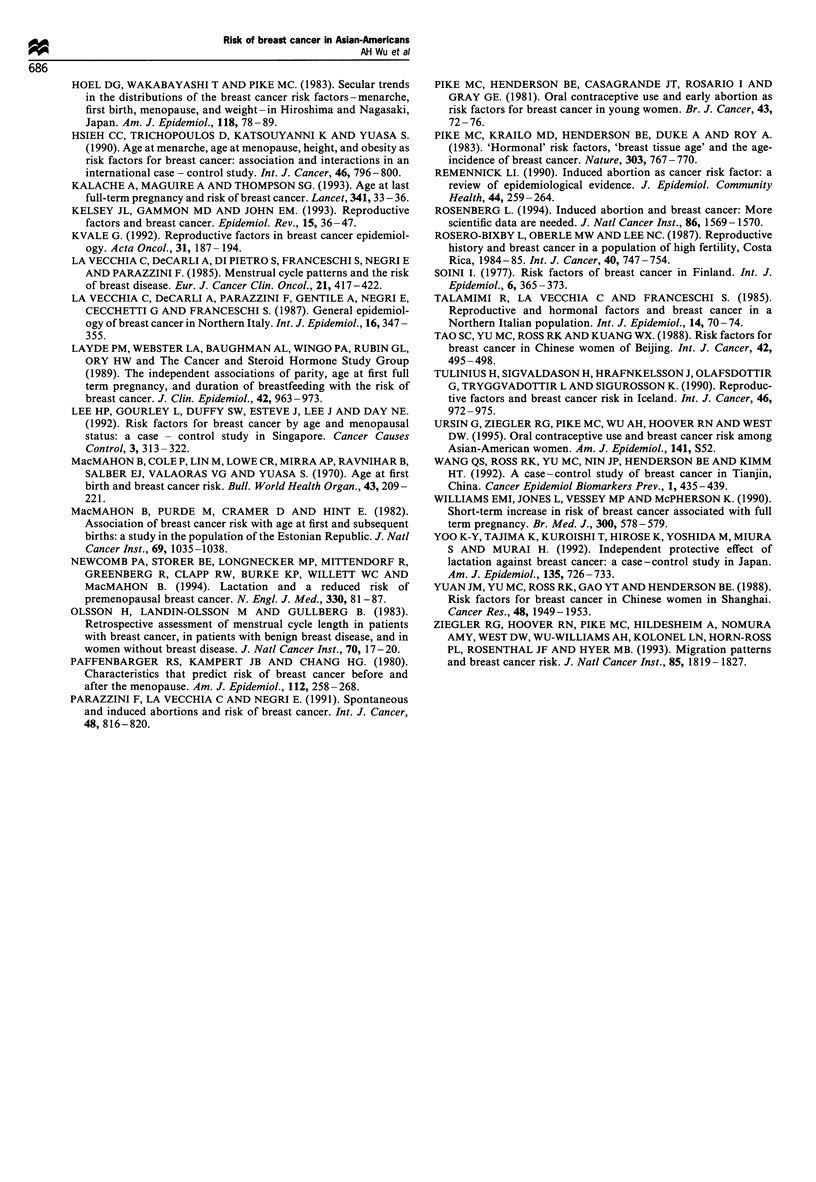# Menstrual and reproductive factors and risk of breast cancer in Asian-Americans.

**DOI:** 10.1038/bjc.1996.118

**Published:** 1996-03

**Authors:** A. H. Wu, R. G. Ziegler, M. C. Pike, A. M. Nomura, D. W. West, L. N. Kolonel, P. L. Horn-Ross, J. F. Rosenthal, R. N. Hoover

**Affiliations:** Department of Preventive Medicine, University of Southern California, Los Angeles, 90033, USA.

## Abstract

We conducted a population-based case-control study of breast cancer among Chinese-, Japanese- and Filipino-American women in Los Angeles County Metropolitan Statistical Area (MSA), San Francisco-Oakland MSA and Oahu, Hawaii. One objective of the study was to quantify breast cancer risks in relation to menstrual and reproductive histories in migrant and US-born Asian-Americans and to establish whether the gradient of risk in Asian-Americans can be explained by these factors. Using a common study design and questionnaire in the three study areas, we successfully conducted in-person interviews with 597 Asian-American women diagnosed with incident, primary breast cancer during the period 1983-87 (70% of those eligible) and 966 population-based controls (75% of those eligible). Controls were matched to cases on age, ethnicity and area of residence. In the present analysis, which included 492 cases and 768 controls, we observed a statistically non-significant 4% reduction in risk of breast cancer with each year delay in onset of menstruation. Independent of age at menarche risk of breast cancer was lower (odds ratio; OR=0.77) among women with menstrual cycles greater than 29 days. Parous Asian-American women showed a significantly lower risk of breast cancer then nulliparous women (OR=0.54). An increasing number of livebirths and a decreasing age at first livebirth were both associated with a lower risk of breast cancer, although the effect of number of livebirths was no longer significant after adjustment for age at first livebirth. Women with a pregnancy (spontaneous or induced abortions) but no livebirth had a statistically non-significant increased risk (OR=1.84), but there was no evidence that one type of abortion was particularly harmful. A positive history of breastfeeding was associated with non-significantly lower risk of breast cancer (OR=.78). There are several notable differences in the menstrual and reproductive factors between Asian-Americans in this study and published data on US whites. US-born Asian Americans had an average age at menarche of 12.12 years-no older than has been found in comparable studies of US whites, but 1.4 years earlier than Asian women who migrated to the US. Asian-American women, particularly those born in the US and those who migrated before age 36, also had a later age at first birth and fewer livebirths than US whites. A slightly higher proportion of Asian-American women breastfed, compared with US whites. The duration of breastfeeding was similar in US-born Asians and US whites, but was longer in Asian migrants, especially those who migrated at a later age. Menstrual and reproductive factors in Asian-American women are consistent with their breast cancer rates being at least as high as in US whites, and they are. However, the effects of these menstrual and reproductive factors were small and the ORs for migration variables changed only slightly after adjustment for these menstrual and reproductive factors. These results suggest that the lower rates of breast cancer in Asians must be largely as a result of other environmental/lifestyle factors.


British Journal of Cancer (1996) 73, 680-686
© 1996 Stockton Press All rights reserved 0007-0920/96 $12.00

### Menstrual and reproductive factors and risk of breast cancer in Asian-Americans

AH Wu[1], R G Ziegler[2], MC Pike[1], AMY Nomura[3], DW West[4], LN Kolonel[3], PL Horn-Ross[4], JF Rosenthal[5] and RN Hoover[2]

[1]*Department of Preventive Medicine, University of Southern California, Los Angeles, CA 90033, USA;* [2]*Environmental Epidemiology Branch, Division of Cancer Etiology, National Cancer Institute, Bethesda, MD 20892, USA;* [3]*Epidemiology Program, Cancer Research Center of Hawaii, University of Hawaii, Honolulu 96817, USA;* [4]*Northern California Cancer Center, Union City, CA 94587, USA;* [5]*Westat, Inc, Rockville, MD 20892, USA.*

**Summary** We conducted a population-based case–control study of breast cancer among Chinese-, Japanese- and Filipino-American women in Los Angeles County Metropolitan Statistical Area (MSA), San Francisco-Oakland MSA and Oahu, Hawaii. One objective of the study was to quantify breast cancer risks in relation to menstrual and reproductive histories in migrant and US-born Asian-Americans and to establish whether the gradient of risk in Asian-Americans can be explained by these factors. Using a common study design and questionnaire in the three study areas, we successfully conducted in-person interviews with 597 Asian-American women diagnosed with incident, primary breast cancer during the period 1983–87 (70% of those eligible) and 966 population-based controls (75% of those eligible). Controls were matched to cases on age, ethnicity and area of residence. In the present analysis, which included 492 cases and 768 controls, we observed a statistically non-significant 4% reduction in risk of breast cancer with each year delay in onset of menstruation. Independent of age at menarche risk of breast cancer was lower (odds ratio; OR = 0.77) among women with menstrual cycles greater than 29 days. Parous Asian-American women showed a significantly lower risk of breast cancer than nulliparous women (OR = 0.54). An increasing number of livebirths and a decreasing age at first livebirth were both associated with a lower risk of breast cancer, although the effect of number of livebirths was no longer significant after adjustment for age at first livebirth. Women with a pregnancy (spontaneous or induced abortions) but no livebirth had a statistically non-significant increased risk (OR = 1.84), but there was no evidence that one type of abortion was particularly harmful. A positive history of breastfeeding was associated with non-significantly lower risk of breast cancer (OR = 0.78). There are several notable differences in the menstrual and reproductive factors between Asian-Americans in this study and published data on US whites. US-born Asian-Americans had an average age at menarche of 12.2 years–no older than has been found in comparable studies of US whites, but 1.4 years earlier than Asian women who migrated to the US. Asian-American women, particularly those born in the US and those who migrated before age 36, also had a later age at first birth and fewer livebirths than US whites. A slightly higher proportion of Asian-American women breastfed, compared with US whites. The duration of breastfeeding was similar in US-born Asians and US whites, but was longer in Asian migrants, especially those who migrated at a later age. Menstrual and reproductive factors in Asian-American women are consistent with their breast cancer rates being at least as high as in US whites, and they are. However, the effects of these menstrual and reproductive factors were small and the ORs for migration variables changed only slightly after adjustment for these menstrual and reproductive factors. These results suggest that the lower rates of breast cancer in Asians must be largely as a result of other environmental/lifestyle factors.

**Keywords:** breast cancer; menstrual factor; reproductive factor; Asian-Americans; migrants; US-born

The importance of menstrual and reproductive factors (age at menarche, age at first birth, parity, age at and type of menopause) as determinants of a woman's risk of breast cancer is well established (MacMahon *et al.*, 1970; Henderson *et al.*, 1984; Kelsey *et al.*, 1993). These factors have been found to be important in high-risk Western populations (Kvale, 1992) and in low-risk Asian groups (Tao *et al.*, 1988; Yuan *et al.*, 1988; Wang *et al.*, 1992), although the magnitude of risks and the relative importance of specific factors have varied in different studies. In studies conducted to compare age at menarche among Japanese in Hiroshima and Nagasaki with those of US whites this risk factor was found to explain about one-third of the 4- to 6-fold difference in breast cancer incidence rates between Japanese and US whites in the 1970s (Hoel *et al.*, 1983; Pike *et al.*, 1983). The extent to which changes in menstrual and reproductive factors can explain the increase in incidence rates of breast cancer in Asian-Americans, including those migrating from Asia and those born in the US, has not been investigated previously. This study consisted of women who differed in several ways from women in earlier studies of Asian-Americans: three Asian ethnic groups were included, one of which (Filipinos) has not previously been examined. In addition, more of the subjects were Western-born (including third- and fourth-generation Asian-Americans) and the Asian migrants were a heterogeneous group from urban and rural areas in Asia. In this report, we examine the role of menstrual and reproductive factors in a population-based case–control study of breast cancer in Chinese-, Japanese- and Filipino-Americans living in the San Francisco-Oakland Metropolitan Statistical Area (MSA), Los Angeles MSA or Oahu, Hawaii.

### Methods

Study methods have been described in detail previously (Ziegler *et al.*, 1993). Briefly, this study included all women of Chinese, Japanese or Filipino ethnicity who were diagnosed with histologically confirmed, first primary breast cancer (ICD-O 174) at ages 20–55 years in the San Francisco-Oakland MSA, the Los Angeles MSA or Oahu, Hawaii, at diagnosis, during the period 1 April 1983 to 30 June 1987. In the California study areas, population controls were selected by random-digit dialling. In Hawaii, population controls were selected with the Health Surveillance Program of the Hawaii

Correspondence: AH Wu, University of Southern California, 1420 San Pablo Street, PMB B300, Los Angeles, CA 90033, USA
Received 9 February 1995; revised 21 September 1995; accepted 28 September 1995

Department of Health, which annually samples 2% of the households in Hawaii. Controls were matched to cases on age (5 year age groups), ethnicity and study area, with the aim of interviewing twice as many controls as cases. Frequency matching was used in California, whereas controls were individually matched in Hawaii. A case or control subject had to be at least 50% Chinese, Japanese or Filipina, or 50% a mixture of these ethnicities. In-person interviews were conducted with the subjects using a structured questionnaire in the language they preferred (English, Chinese or Japanese). All interviews were conducted between August 1985 and February 1989. We completed interviews with 70% of eligible cases and 75% of eligible controls. The participation rates for cases (68–75%) and controls (70–79%) were similar in the three study areas; each centre interviewed approximately one-third of the study subjects (Ziegler *et al.*, 1993).

Our in-person interview elicited information on each woman's menstrual and reproductive history. To assesss menstrual history of subjects we asked the age when they had their first menstrual period, age when their menstrual periods became established at regular intervals (i.e. there was a predictable amount of time between menstrual periods), and the number of days between the start of each period once they became regular. Subjects were asked the total number of pregnancies they had. For each pregnancy, the outcome of the pregnancy (i.e. livebirth, stillbirth, induced abortion, spontaenous abortion and tubal or ectopic pregnancy), the length of the pregnancy, the year when the pregnancy ended, whether the baby was breastfed and the duration of breastfeeding were asked.

For each menstrual and reproductive variable, odds ratios (ORs, relative risk estimates), their corresponding 95% confidence intervals (95% CI) and two-sided statistical significance levels (*P*-values) were calculated. Tests for linear trend were performed on all continuous variables. Unconditional logistic regression methods were used with single variables, as well as for multivariate analysis (Breslow and Day, 1980). The ORs were first adjusted for the variables used in matching, i.e. ethnicity, study area and age (in 5 year age groups) (this adjustment must be made in all analyses because of the design of the study). Then, the ORs were additionally adjusted for migration history, which we reported to strongly affect the risk of breast cancer (Ziegler *et al.*, 1993). The adjustment for migration history was made so that the ORs directly associated with menstrual and reproductive factors could be determined. In most instances the ORs were attenuated only slightly with adjustment for migration and we present these migration-adjusted ORs in Tables II–IV as a conservative estimate of the strength of the relative risks. For completeness, both sets of ORs (with and without adjustment for migration history) are shown in the summary table on multivariate models (Table V). The migration variables were birthplace of the subject, the West or the East (West included USA, Canada, western and central Europe, the former USSR, Australia and New Zealand, and East included Asia, Southeast Asia, the Malaysian Peninsula, Singapore, India and countries in the Southwest Pacific excluding Australia and New Zealand). For subjects born in the East, they were further categorised by whether they always lived in urban or rural areas in the East and by years of residence in the West ($\leqslant 7$ *vs* 8+years) (Ziegler *et al.*, 1993). In addition, we included birthplace (East or West) of the subject's maternal grandmother. The effect on risk of the birthplaces of the subject's parents and all other grandparents was not significant after adjustment for the place of birth of the subject's maternal grandmother (Ziegler *et al.*, 1993). Excluded from the analyses were two women for whom their place of birth was not known; 27 women who were born in the West but their maternal grandmothers' place of birth was not known; 55 women who had been in the West for 1 year or less at the time of cancer diagnosis or interview; 55 women who were born in the West but had lived in the East; 80 women who were born in the East and had at least three moves between the East and West; 66 women who lived in both urban and rural areas while in the East and; 18 women for whom we did not have complete information on menstrual and reproductive history.

To evaluate the fit of various statistical models we calculated twice the difference in the log-likelihoods (equivalent to difference of chi-squares) and the associated difference in degrees of freedom between any two models being compared with adjustment for demographic variables and migration variables. The statistical significance of the difference in the fit of two models is calculated from the upper tail of the chi-square distribution (*P*-value).

**Table I** Comparison of menstrual and reproductive characteristics of Asian-American breast cancer cases and controls

| | *All subjects*[a] | *Born in West*[b] | *Born in East*[c] |
|---|---|---|---|
| Number of controls | 768 | 355 | 413 |
| Number of cases | 492 | 248 | 244 |
| Mean age at menarche | | | |
| Controls | 13.0 | 12.2 | 13.6 |
| Cases | 12.9 | 12.2 | 13.5 |
| Number (%) never pregnant | | | |
| Controls | 94(12.2) | 40(11.3) | 54(13.1) |
| Cases | 91(18.5) | 48(19.4) | 43(17.6) |
| Mean number of pregnancies (among gravid women) | | | |
| Controls | 3.2 | 3.1 | 3.4 |
| Cases | 3.1 | 2.9 | 3.2 |
| Number (%) with no livebirth | | | |
| Controls | 112(14.6) | 49(13.8) | 63(15.3) |
| Cases | 119(24.2) | 67(27.0) | 52(21.3) |
| Mean number of livebirths (among parous women) | | | |
| Controls | 2.7 | 2.7 | 2.8 |
| Cases | 2.6 | 2.6 | 2.6 |
| Mean age at first livebirth (among parous women) | | | |
| Controls | 26.1 | 25.5 | 26.6 |
| Cases | 27.1 | 26.0 | 28.1 |
| Percentage ever breastfed[d] (among parous women) | | | |
| Controls | 54.9 | 53.8 | 55.9 |
| Cases | 43.1 | 40.6 | 45.5 |
| Mean number weeks of breast feeding (among parous women) | | | |
| Controls | 42.2 | 28.0 | 54.7 |
| Cases | 35.0 | 21.4 | 47.8 |

[a]See Methods for exclusions. [b]US, Canada, western and central Europe, the former USSR, Australia and New Zealand. [c]China, Japan, Phillipines, Taiwan, Hong Kong, Macau, Southeast Asia, the Malaysian Peninsula, Singapore, India and countries in the Southwest Pacific excluding Australia and New Zealand. [d]For at least one month.

### Results

In this study 70% of the women were premenopausal (i.e. at diagnosis for cases or at the assigned diagnosis date for controls (Ziegler *et al.*, 1993)). The mean (standard deviation) ages for cases and controls were 45.3 (7.01) and 44.6 (7.84) respectively at diagnosis. A summary of selected menstrual and reproductive variables for all cases and controls, stratified by birthplace, is shown in Table I. Compared with Asian-American women born in the West, Asian women who migrated to the US had later age at menarche, reported slightly more pregnancies and more livebirths (but a slightly greater proportion of nulliparity and a later age at first livebirth) and breastfed for a considerably longer time. There was a strong secular trend by year of birth in average age at menarche in migrant women. Control women born around 1955 (i.e. about age 30 at interview) had an average age at menarche of 13.0; this steadily increased to 14.5 for women born around 1925 (i.e. around age 55 at interview). The results of the case–control comparisons shown in Table I are presented in more detail in Tables II–V.

The associations between menstrual factors and risk of breast cancer are shown in Table II. Although the decline was not completely consistent year by year, and was not formally statistically significant, risk of breast cancer decreased on average 6% for each year that age at menarche was delayed (OR = 0.94, 95% CI = 0.86–1.03). Women who

**Table II** Age at menarche and menstrual cycle length and risk of breast cancer

| Variable | Cases/controls | Adjusted[a] OR | 95% CI |
|---|---|---|---|
| Age at menarche (years) | | | |
| ⩽12 | 239/336 | 1.00 | |
| 13 - 14 | 180/288 | 0.87 | 0.67 - 1.14 |
| 15+ | 73/144 | 0.69 | 0.48 - 1.00 |
| Per year[b] | | 0.94 | 0.86 - 1.03 |
| Ever regular periods[c] | | | |
| Yes | 461/720 | 1.00 | |
| No | 30/48 | 0.80 | 0.49 - 1.31 |
| Age at regular periods (years) | | | |
| ⩽12 | 156/221 | 1.00 | |
| 13 - 14 | 154/243 | 0.91 | 0.67 - 1.23 |
| 15+[d] | 173/295 | 0.77 | 0.57 - 1.04 |
| Cycle length (days)[e] | | | |
| ⩽26 | 55/88 | 0.87 | 0.59 - 1.29 |
| 27 - 29 | 278/378 | 1.00 | |
| 30+ | 118/245 | 0.72 | 0.54 - 0.95 |

[a]Adjusts for age (in 5 year age groups), area, ethnicity and migration history (see Methods section). [b]With age at menarche in single years; 15 subjects were missing on age at menarche. [c]Nineteen subjects were missing on ever had regular cycles. [d]Including 30 cases and 48 controls who were never regular. [e]Among subjects with regular periods; 19 subjects were missing on cycle length.

**Table III** Pregnancy history and risk of breast cancer

| Variable | Cases/controls | Adjusted[a] OR | 95% CI |
|---|---|---|---|
| Ever pregnant | | | |
| No | 91/94 | 1.00 | |
| Yes | 401/674 | 0.57 | 0.41–0.80 |
| Never pregnant | 91/94 | 1.00 | |
| Pregnant, 0 LB | 28/18 | 1.72 | 0.87–3.41 |
| Pregnant, 1+ LB | 373/656 | 0.54 | 0.39–0.75 |
| Never pregnant | 91/94 | 1.00 | |
| Pregnant, 0 LB | 28/18 | 1.79 | 0.90–3.56 |
| LB = 1 | 65/114 | 0.59 | 0.38–0.92 |
| 2 | 144/238 | 0.59 | 0.41–0.86 |
| 3 | 104/166 | 0.55 | 0.37–0.83 |
| 4 | 33/74 | 0.37 | 0.22–0.63 |
| 5+ | 27/64 | 0.32 | 0.18–0.57 |
| Never pregnant | 91/94 | 1.00 | |
| Pregnant, 0 LB | 28/18 | 1.80 | 0.90–3.57 |
| LB = 1 | 65/114 | 0.67 | 0.46–0.96 |
| Per additional LB | 308/542 | 0.87 | 0.78–0.96 |
| Never pregnant | 91/94 | 1.00 | |
| Pregnant, 0 LB | 28/18 | 1.77 | 0.89–3.52 |
| Age at first livebirth (years) | | | |
| ⩽19 | 14/40 | 0.24 | 0.12–0.50 |
| 20 - 24 | 112/227 | 0.44 | 0.30–0.66 |
| 25 - 29 | 143/238 | 0.58 | 0.40–0.84 |
| 30 - 34 | 70/117 | 0.58 | 0.37–0.89 |
| 35+ | 34/34 | 1.06 | 0.59–1.90 |
| Never pregnant | 91/94 | 1.00 | |
| Pregnant, 0 LB | 28/18 | 1.76 | 0.89–3.51 |
| AFLB 25 - 29 | 143/238 | 0.54 | 0.39–0.76 |
| Per 5 year change in FLB | 230/418 | 1.28 | 1.12–1.48 |
| Never pregnant | 91/94 | 1.00 | |
| Pregnant, 0 LB | 28/18 | 1.80 | 0.91–3.58 |
| AFLB 25 - 29 | 143/238 | 0.62 | 0.43–0.89 |
| Per 5 year change in FLB | | 1.22 | 1.05–1.42 |
| Per additional LB | | 0.92 | 0.82–1.03 |

AFLB, age at first livebirth; FLB, first livebirth; LB, livebirth. [a]Adjusts for age (in 5 year age groups), area, ethnicity and migration history (see Methods section).

**Table IV** Pregnancy outcome and risk of breast cancer

| Variable | Cases/controls | Adjusted OR[a] | 95% CI |
|---|---|---|---|
| Never pregnant | 91/94 | 1.00 | |
| No livebirth (LB), 1+ abortions[b] | 28/18 | 1.72 | 0.87–3.40 |
| No LB, spontaneous abortions alone | 12/7 | 1.34 | 0.53–3.36 |
| No LB, induced abortions alone | 12/10 | 1.92 | 0.70–5.30 |
| No LB, at least one spontaneous abortion[c] | 16/8 | 2.20 | 0.87–5.55 |
| No LB, at least one induced abortion[d] | 16/11 | 1.60 | 0.68–3.74 |
| 1+LB | 373/656 | 0.54 | 0.39–0.75 |
| Never pregnant | 91/94 | 1.00 | |
| No LB, 1+ abortions[b] | 28/18 | 1.72 | 0.87–3.40 |
| 1+ LB, no abortion | 225/430 | 0.50 | 0.35–0.71 |
| 1+ LB, abortions only before 1st LB | 28/53 | 0.53 | 0.30–0.92 |
| 1+ LB, abortions only after 1st LB | 104/157 | 0.61 | 0.41–0.91 |
| 1+ LB, abortions before and after 1st LB | 17/17 | 0.89 | 0.42–1.92 |

[a]Adjusts for age (in 5 year age groups), area, ethnicity and migration history (see Methods section). [b]Includes spontaneous and induced abortions; 12 cases and seven controls had spontaneous abortions alone; 12 cases and ten controls had induced abortions alone; four cases and one control had both spontaneous and induced abortions. [c]Includes subjects with both spontaneous and induced abortions and those with spontaneous abortions alone. [d]Includes subjects with both spontaneous and induced abortions and those with induced abortions alone.

never had regular periods showed a lower risk of breast cancer than women who had regular periods and women who had late onset of regular periods also showed a lower risk of breast cancer than women who reported having regular periods at an earlier age (Table II). However, in this study, there was little added protection associated with never having regular periods (OR = 0.84, $P = 0.48$) or with later age at regular periods (ORs were 1.00 and 0.90 respectively, for age at regular periods at 13–14, 15+ compared with ⩽12; $P = 0.75$) when age at menarche was accounted for. Menstrual cycle length was available on subjects who reported they had regular periods. Compared with women with a menstrual cycle length of 27–29 days women with longer cycles showed a statistically significant reduction in risk (OR = 0.72), and women with shorter cycles showed a smaller, and statistically non-significant reduction in risk (OR = 0.87). The ORs for age at menarche and cycle length were not noticeably altered by adjustment for each other (Table V).

A higher percentage of cases (18.5%) than controls (12.2%) had never been pregnant; this translated into an OR of 0.57 for ever being pregnant (Table III). However, the protection was observed only in women who had at least one livebirth. Women who had been pregnant but had no livebirths experienced a higher risk of breast cancer compared with those who had never been pregnant, although the effect was based on small numbers and was not statistically significant (OR = 1.72, 95% CI = 0.87–3.40) (see Table IV). Relative to women who were never pregnant, a single livebirth was associated with a 33% reduction in risk (OR = 0.67) and each additional livebirth was associated with a further 13% reduction in risk (OR = 0.87; $P = 0.005$). Age at first livebirth also had a significant effect on breast cancer risk. Women with first livebirth before age 20 had an OR of 0.24 compared with women who had never been pregnant. Women with later ages at first livebirth had increasing risks of breast cancer compared with women whose first livebirth was before age 20, and women whose first livebirth was after age 35 showed a risk similar to never pregnant women. Among women with at least one livebirth, the risk for breast cancer increased 28% ($P = 0.0004$) for each 5 years that a first livebirth was delayed after ages 25–29. The effect of age at first livebirth was greater than the effect of number of livebirths; and number of livebirths had only a small effect after fitting age at first livebirth ($P = 0.29$). However, the OR (per livebirth) for number of livebirths only changed from

**Table V** Multivariate analysis of menstrual and reproductive factors and risk of breast cancer

| *Variable* | *Adjusted OR*[a] | *95% CI* | *Adjusted OR*[b] | *95% CI* |
|---|---|---|---|---|
| Never pregnant | 1.00 | | 1.00 | |
| Pregnant, 0 LB | 1.92 | 0.96–3.86 | 1.84 | 0.91–3.70 |
| AFLB 25–29 | 0.78 | 0.52–1.17 | 0.76 | 0.50–1.15 |
| Per 5 year change in FLB | 1.20 | 1.03–1.41 | 1.24 | 1.06–1.45 |
| Per additional LB | 0.94 | 0.84–1.05 | 0.94 | 0.84–1.06 |
| Breastfeeding (⩾1 month) *vs* no | 0.74 | 0.56–0.98 | 0.78 | 0.58–1.04 |
| Cycle length (days)[c] | | | | |
| ⩽26 | 0.90 | 0.61–1.32 | 0.90 | 0.61–1.34 |
| 30+ | 0.76 | 0.57–1.00 | 0.77 | 0.58–1.02 |
| Menarche per year after age 11[d] | 0.92 | 0.84–1.00 | 0.96 | 0.87–1.05 |

[a]Adjusts for age (in 5 year age groups), area and ethnicity. [b]Adjusts for age (in 5 year age groups), area, ethnicity, and migration history (see Methods section). [c]Compared with cycle length of 27–29 days. [d]Compared with age at menarche at 11 years or younger. AFLB, age at first livebirth; FLB, first livebirth; LB, livebirth.

**Table VI** Distribution (%) of menstrual and reproductive factors among Asian[a], Asian-American[b] and US white controls in selected studies

| *Area* | *Shanghai Chinese* | *Tianjin Chinese* | *Singapore Chinese* | *California and Hawaii Asian-Americans* | | | *USA US whites* | |
|---|---|---|---|---|---|---|---|---|
| | | | | *36+*[b] | *<36*[b] | *US-born* | | |
| Ref. | Yuan (1988) | Wang (1992) | Lee (1992) | Current study | | | Layde (1989) | Newcomb (1994) |
| Age range | 20–69 | ⩽55 | 24–88 | 20–55 | | | 20–55 | <75 |
| Age at menarche | | | | | | | | |
| <12 | 8 | 12 | 25 | 25 | 29 | 62 | NA[c] | 39 |
| 13–14 | 33 | 36 | 38 | 37 | 44 | 32 | NA | 47 |
| 15+ | 59 | 52 | 37 | 37 | 27 | 6 | 11 | 14 |
| Number of livebirths | | | | | | | | |
| 0 | 9 | 10 | 11 | 17 | 14 | 14 | 13 | 14 |
| 1 | 16 | 17 | 10 | 10 | 16 | 15 | 11 | 10 |
| 2–3 | 37 | 50 | 38 | 38 | 59 | 51 | 48 | 48 |
| 4+ | 38 | 23 | 41 | 35 | 10 | 19 | 28 | 28 |
| Age at first livebirth | | | | | | | | |
| ⩽19 | 19 | 12 | 21 | 10 | 5 | 6 | 26 | 17 |
| 20–24 | 47 | 42 | 39 | 43 | 27 | 38 | 40 | 49 |
| 25–29 | 27 | 38 | 29 | 35 | 35 | 38 | 28 | 25 |
| 30+ | 7 | 8 | 11 | 12 | 33 | 18 | 6 | 9 |
| History of lactation | | | | | | | | |
| Yes | 92 | 95 | 53 | 76 | 62 | 62 | 54 | 55 |
| ⩽1 year | NA | NA | 27 | 32 | 44 | 50 | 40 | 43 |
| >1 year | NA | NA | 26 | 44 | 18 | 12 | 14 | 12 |
| 1–3 years | 51 | 48 | NA | 54 | 59 | 59 | NA | NA |
| >3 years | 41 | 47 | NA | 22 | 3 | 3 | NA | NA |

[a] The studies chosen included traditional Asian women who lived in urban areas in China but had experienced little Western influence at the time when the studies were conducted (Yuan *et al.*, 1988; Wang *et al.*, 1992) and Asian women who lived in Singapore and may have been more Westernised (Lee *et al.*, 1992). [b] We have divided the controls in our study into US-born, and into migrants who moved to the US at a young age (<36 years; early migrants) or as older women (⩾36 years; late migrants). The latter division was based on our observation that breast cancer risk was decreased in women migrating to the West at 36+ years of age (Ziegler *et al.*, 1993). [c] The distribution for ⩽11, 12–14, 15+ for menarche age was 22, 67 and 11 per cent respectively. NA, not avaliable.

0.87 to 0.92; and we decided to include both variables in all further analyses (Table III). The results could be adequately fitted by single linear terms for age at first livebirth and number of livebirths (after the first).

As we noted above, an abortion never followed by a livebirth was associated with an OR of 1.72 ($P$=0.09) compared with never having been pregnant. This increased risk among women who never had a livebirth existed for both spontaneous and induced abortions but the increased risk was not statistically significant for spontaneous abortions alone, induced abortions alone, at least one spontaneous abortion, at least one induced abortion or any abortion (spontaneous or induced) (Table IV). There was no significant increased risk in relation to abortions among women who had at least one livebirth. Specifically, among women who had at least one livebirth, there was a small, statistically non-significant increase in risk (OR=1.20, 95% CI=0.92–1.58) in women who had an abortion compared with women with no abortions. The risk of breast cancer was still below 1.0 in each subgroup of women when we examined the association by timing of the abortion, i.e. before a first livebirth, after a first livebirth, or before and after a first livebirth (Table IV).

A positive history of breastfeeding for at least 1 month was associated with a lower risk of breast cancer (OR=0.73, 95% CI=0.56–0.95) (data not shown) but there was no statistically significant trend of decreasing risk with increasing number of children breastfed or with increasing duration of breastfeeding (manuscript in preparation). However, breastfeeding for less than 1 month had no protective effect (OR=1.10).

In further analyses, the role of pregnancy history (never pregnant, ever pregnant but no livebirths, one or more livebirths), age at first livebirth, ever breastfed for ⩾1 month, age at menarche and menstrual cycle length were examined simultaneously in a multivariate model (Table V). The risk estimates were weakened somewhat but each of the variables continued to influence risk of breast cancer. The associations reported in Table V showed no appreciable change when age and type of menopause and use of oestrogen replacement therapy were considered in the multivariate regression analysis. These results were also observed in stratified analyses restricted to premenopausal women (manuscript on the influence of menopause is in preparation). The use of and the duration of use of oral contraceptives were not associated with risk of breast cancer in this population (Ursin *et al.*, 1995). These associations were generally similar in analyses conducted within each of the three Asian ethnic groups.

The above results on menstrual and reproductive factors were observed after adjustment for migration history. We also evaluated whether these menstrual and reproductive patterns could account for the 6-fold gradient in risk of breast cancer in Asian-Americans by migration patterns as we noted previously (Ziegler *et al.*, 1993). In Table V two columns of ORs are shown for the various menstrual and reproductive factors; the first column without adjustment for migration factors, and the second column with such adjustment. Relative to Asian-American women who had always lived in the West the respective ORs were 0.20 for always lived in rural communities in the East and for ⩽7 years in the West, 0.67 for always lived in rural communities in the East and for 8+ years in the West, 0.48 for always lived in urban communities in the East and ⩽7 years in the West, and 0.75 for always lived in urban communities in the East and 8+ years in the West. The ORs for migration were only slightly changed, becoming 0.25, 0.69, 0.48 and 0.78 respectively, after adjustment for these menstrual and reproductive factors (Table V).

### Discussion

The main objective of this analysis was to determine the role of menstrual and reproductive factors in the aetiology of breast cancer in younger Asian-American women. Although the recall of menstrual and reproductive factors may be less difficult for younger women (presumably resulting in less random misclassification), the associations we observed were generally weak. As in most other case–control studies of breast cancer, which rely on self-reported information, no attempt was made to assess the reliability of the data collected in this study.

Our finding of a 4% reduction in the risk of breast cancer with each year delay in onset of menstruation (Table V) is consistent with previous findings (MacMahon *et al.*, 1970; Hsieh *et al.*, 1990). Irregular menstrual cycles (La Vecchia *et al.*, 1985, 1987; Soini, 1977) or delay in establishment of regular menses, independent of age at menarche, decreased the risk in some studies (Henderson *et al.*, 1981). However, in this study, the additional protection associated with never achieving regular menses or with late age at regular menses was small and was not statistically significant after adjustment for age at menarche.

Although our question on cycle length was limited to one time period and length of cycles may change with age, the data in this study (Table II) suggested a U-shape relationship between length of regular menstrual cycle and risk of breast cancer. We investigated this further by examining our data by single day of cycle length. Our results showed that compared with women with a 28 day cycle, women with cycles of 29, 30, 31 or 32 days or longer showed ORs of 1.06, 0.69, 0.98 and 0.81 respectively, whereas women with cycles of 27, 26, 25 or 24 days or shorter showed ORs of 1.39, 1.15, 1.25 and 0.54 respectively. One interpretation of this result is that women with very short cycles (i.e. fewer than 25 days) ovulated infrequently and that these women as well as women with long cycles have a reduction in risk. Women with cycles of 25–27 days may be at increased risk. In the study by Olsson *et al.* (1983), women with long cycles (>30 days) were found to be at reduced risk of breast cancer, but other studies have not found this (Yuan *et al.*, 1988; La Vecchia *et al.*, 1987). Elevated risks of breast cancer in relation to short menstrual cycles have been reported in some studies (Yuan *et al.*, 1988; La Vecchia *et al.*, 1987; Olsson *et al.*, 1983); the definition of short menstrual cycles ranged from less than 21 days (Olsson *et al.*, 1983) to less than 26 days (La Vecchia *et al.*, 1987) in these studies.

The increased risk of breast cancer with earlier age at menarche is thought to be due to extended exposure to oestrogens and possibly progesterone. There is considerable data suggesting that early menarche is associated with early onset of regular cycles, and presumably onset of regular ovulation (MacMahon *et al.*, 1982; Apter and Vihko, 1984; Henderson *et al.*, 1985). Early onset of menarche is thus associated with longer duration of exposure to ovarian hormone levels associated with ovulation. There is also support from some studies (Apter and Vihko, 1984; Apter *et al.*, 1989) that earlier onset of regular menstrual cycles is associated with long-lasting higher oestrogen levels and lower sex-hormone globulin binding capacity (SHBG) (Apter *et al.*, 1989), although this has not been consistently found (Bernstein *et al.*, 1991).

Nulliparous Asian-American women have a significantly elevated risk of breast cancer compared with parous women as a group, consistent with results observed in previous studies conducted in high-risk (Paffenbarger *et al.*, 1980; Brinton *et al.*, 1983; Layde *et al.*, 1989) and intermediate-risk (La Vecchia *et al.*, 1987; Soini, 1977; Talamini *et al.*, 1985; Rosero-Bixby *et al.*, 1987) western countries and in low-risk eastern countries (Tao *et al.*, 1988; Yuan *et al.*, 1988; Wang *et al.*, 1992; Hlaing and Myint, 1978; Yoo *et al.*, 1992). Among parous women, an increasing number of livebirths was associated with decreasing risk while increasing age at first livebirth was positively associated with risk. The decrease in risk with increasing number of livebirths was reduced from 13% (per livebirth) to 8% and was no longer statistically significant, after adjustment for age at first livebirth (Table III). The relative importance of parity and age at first birth on risk of breast cancer has varied in different studies. Although the discrepancies between studies remain unexplained (Kelsey et al., 1993; Kvale, 1992), it is important to note that the effects of these variables may not be directly comparable since the units used to express parity and age at first birth differ. Some previous studies suggest that an effect of age at first birth is stronger in younger women (<55 years) (Layde et al., 1989; Ewertz et al., 1990; Tulinius et al., 1990); our data support this. In our data, the increased risks associated with older age at first birth were more apparent in women under the age of 50 than in those over age 50, whereas the protective effect of number of livebirths was evident mainly in women over age 50 (data not shown). The differences in risk estimates in older and younger women were, however, not statistically significant for either reproductive variable. An increased risk with decreasing interval since last term birth (Bruzzi et al., 1988; Williams et al., 1990) and with increasing age at last full-term pregnancy (Kalache et al., 1993) has been suggested. Results from this study do not show a consistent association between years since last term birth and risk of breast cancer (data not shown).

In this study, compared with women who had never been pregnant, women who had a spontaneous or induced abortion that was never followed by a livebirth showed a statistically non-significant higher risk (OR = 1.72) of breast cancer (Table IV). Among women who had at least one livebirth there was also no significant increased risk associated with spontaneous or induced abortion, regardless of whether the abortion occurred before and/or after the first livebirth. Since the first report of an increased risk of breast cancer in association with abortion (Pike et al., 1981), some 30 studies have evaluated this relationship and have failed to reach a consensus (Pike et al., 1981; Parazzini et al., 1991; Remennick, 1990; Daling et al., 1994; Rosenberg, 1994). In a recent study of women younger than 45 years, Daling et al. (1994) reported about a 50% increased risk for induced abortion whereas no increased risk was associated with spontaneous abortion. The present study suggests a small increased risk with both spontaneous and induced abortion; neither increase was statistically significant. The study by Daling et al. (1994) also suggested that the risk was particularly elevated among subjects who had induced abortions at a young age (<18 years) or at older ages (30+ years) and if the abortion took place after 8 weeks' gestation. None of the cases and one control had induced abortions at ages <18 years whereas the OR was 1.75 for having an induced abortion at 30+ years compared with at ages 18–30 years. The risks for induced abortion occurring at 1–8 weeks and beyond 8 weeks of gestation in this study were 1.79 and 1.14 respectively.

Table VI summarises the distribution of menstrual and reproductive factors for population controls from selected studies conducted in Asia during the 1980s (columns 1–3), for Asian-Americans in this study (columns 4–6) and for US white women (column 7–8). There are several notable differences between Asian-American women and Asian women. Some 62% of US-born Asian-American women had menarche at age 12 or younger, compared with 8–12% of Chinese in China, 25% of Chinese in Singapore and 25–29% in Asian migrants to the US. This 62% figure may even exceed that of US whites (39%). The percentage of nulliparous Asian-Americans is similar to US whites and is somewhat higher than that for Asians. Of note is the considerable delay in childbearing and fewer number of children of early migrants and US-born Asian-American women compared with Asians, late migrants and US whites. The lactation pattern of late migrants and US born Asian-Americans was intermediate between those of low-risk women in China and high-risk US whites. Thus, the increase in risk of breast cancer in Asian-Americans may be explained in part by the earlier age at menarche among US-born subjects, by delay of childbearing and the tendency to have fewer children, and by either not breastfeeding or breastfeeding only for a short period of time.

Our study showed, however, that the above effects are small. The menstrual and reproductive factors do not explain the gradient in risk in Asian-Americans. The ORs for the migration variables were only slightly altered by inclusion of menstrual and reproductive factors in the statistical model. This shows that the striking gradient of risk in Asian-Americans, which spans the difference between rates in Asia and rates in US whites, cannot be explained by altered reproductive factors. The major differences in breast cancer between Asian migrants and US-born Asians seem to be due to other factors that differ between the groups, possibly differences in diet and in physical activity. The menstrual and reproductive factors in US-born Asian-American women are similar to those of US whites (Table VI) and suggest, other factors being equal, that their breast cancer rates might be similar to those in US whites, and they are (Ziegler et al., 1993). Ethnicity-related genetic differences between Asians and US whites would therefore appear to have only a minor role in explaining any differences between Asian and Western breast cancer rates. We need to concentrate our efforts in defining the environmental/lifestyle factors that must be the major explanation for the substantially lower breast cancer rates in Asian women.

**Acknowledgements**
We thank Peggy Wan for technical help.

### References

APTER D AND VIHKO R. (1984). Endocrine characteristics of adolescent menstrual cycles: impact of early menarche. *J. Steroid. Biochem.*, **20**, 231–236.

APTER D, REINILA M AND VIHKO R. (1989). Some endocrine characteristics of early menarche, a risk factor for breast cancer, are preserved into adulthood. *Int. J. Cancer*, **44**, 783–787.

BERNSTEIN L, PIKE MC, ROSS RK AND HENDERSON BE. (1991). Age at menarche and estrogen concentrations of adult women. *Cancer Causes Control*, **2**, 221–225.

BRESLOW NE AND DAY NE. (1980). *Statistical Methods in Cancer Research*, Vol. I. IARC Scientific Publications No. 32. IARC: Lyon.

BRINTON LA, HOOVER R AND FRAUMENI JF. (1983). Reproductive factors in the aetiology of breast cancer. *Br. J. Cancer*, **47**, 757–762.

BRUZZI P, NEGRI E, LA VECCHIA C, DECARLI A, PALLI D, PARAZZINI F AND DEL TURCO MR. (1988). Short term increase in risk of breast cancer after full term pregnancy. *Br. Med. J.*, **297**, 1096–1098.

DALING JR, MALONE KE, VOIGT LF, WHITE E AND WEISS NS. (1994). Risk of breast cancer among young women: Relationship to induced abortion. *J. Natl Cancer Inst.*, **86**, 1584–1592.

EWERTZ M, DUFFY SW, ADAMI HO, KVALE G, LUND E, MEIRIK O, MELLEMGAARD A, SOINI I AND TULINIUS H. (1990). Age at first birth, parity and risk of breast cancer. A meta-analysis of 8 studies from the Nordic countries. *Int. J. Cancer*, **46**, 597–603.

HENDERSON BE, PIKE MC AND CASAGRANDE JT. (1981). Breast cancer and the oestrogen window hypothesis. *Lancet*, **2**, 363–374.

HENDERSON BE, PIKE MC AND ROSS RK. (1984). Epidemiology and risk factors. In *Breast Cancer: Diagnosis and Management*, Bonadonna G. (ed.). Chichester: John Wiley & Sons.

HENDERSON BE, ROSS RK, JUDD HL, KRAILO MD AND PIKE MC. (1985). Do regular ovulatory cycles increase breast cancer risk? *Cancer*, **56**, 1206–1208.

HLAING T AND MYINT TM. (1978). Risk factors of breast cancer in Burma. *Int. J. Cancer*, **21**, 432–437.

HOEL DG, WAKABAYASHI T AND PIKE MC. (1983). Secular trends in the distributions of the breast cancer risk factors – menarche, first birth, menopause, and weight – in Hiroshima and Nagasaki, Japan. *Am. J. Epidemiol.*, **118,** 78 – 89.

HSIEH CC, TRICHOPOULOS D, KATSOUYANNI K AND YUASA S. (1990). Age at menarche, age at menopause, height, and obesity as risk factors for breast cancer: association and interactions in an international case – control study. *Int. J. Cancer*, **46,** 796 – 800.

KALACHE A, MAGUIRE A AND THOMPSON SG. (1993). Age at last full-term pregnancy and risk of breast cancer. *Lancet*, **341,** 33 – 36.

KELSEY JL, GAMMON MD AND JOHN EM. (1993). Reproductive factors and breast cancer. *Epidemiol. Rev.*, **15,** 36 – 47.

KVALE G. (1992). Reproductive factors in breast cancer epidemiology. *Acta Oncol.*, **31,** 187 – 194.

LA VECCHIA C, DeCARLI A, DI PIETRO S, FRANCESCHI S, NEGRI E AND PARAZZINI F. (1985). Menstrual cycle patterns and the risk of breast disease. *Eur. J. Cancer Clin. Oncol.*, **21,** 417 – 422.

LA VECCHIA C, DeCARLI A, PARAZZINI F, GENTILE A, NEGRI E, CECCHETTI G AND FRANCESCHI S. (1987). General epidemiology of breast cancer in Northern Italy. *Int. J. Epidemiol.*, **16,** 347 – 355.

LAYDE PM, WEBSTER LA, BAUGHMAN AL, WINGO PA, RUBIN GL, ORY HW and The Cancer and Steroid Hormone Study Group (1989). The independent associations of parity, age at first full term pregnancy, and duration of breastfeeding with the risk of breast cancer. *J. Clin. Epidemiol.*, **42,** 963 – 973.

LEE HP, GOURLEY L, DUFFY SW, ESTEVE J, LEE J AND DAY NE. (1992). Risk factors for breast cancer by age and menopausal status: a case – control study in Singapore. *Cancer Causes Control*, **3,** 313 – 322.

MacMAHON B, COLE P, LIN M, LOWE CR, MIRRA AP, RAVNIHAR B, SALBER EJ, VALAORAS VG AND YUASA S. (1970). Age at first birth and breast cancer risk. *Bull. World Health Organ.*, **43,** 209 – 221.

MacMAHON B, PURDE M, CRAMER D AND HINT E. (1982). Association of breast cancer risk with age at first and subsequent births: a study in the population of the Estonian Republic. *J. Natl Cancer Inst.*, **69,** 1035 – 1038.

NEWCOMB PA, STORER BE, LONGNECKER MP, MITTENDORF R, GREENBERG R, CLAPP RW, BURKE KP, WILLETT WC AND MacMAHON B. (1994). Lactation and a reduced risk of premenopausal breast cancer. *N. Engl. J. Med.*, **330,** 81 – 87.

OLSSON H, LANDIN-OLSSON M AND GULLBERG B. (1983). Retrospective assessment of menstrual cycle length in patients with breast cancer, in patients with benign breast disease, and in women without breast disease. *J. Natl Cancer Inst.*, **70,** 17 – 20.

PAFFENBARGER RS, KAMPERT JB AND CHANG HG. (1980). Characteristics that predict risk of breast cancer before and after the menopause. *Am. J. Epidemiol.*, **112,** 258 – 268.

PARAZZINI F, LA VECCHIA C AND NEGRI E. (1991). Spontaneous and induced abortions and risk of breast cancer. *Int. J. Cancer*, **48,** 816 – 820.

PIKE MC, HENDERSON BE, CASAGRANDE JT, ROSARIO I AND GRAY GE. (1981). Oral contraceptive use and early abortion as risk factors for breast cancer in young women. *Br. J. Cancer*, **43,** 72 – 76.

PIKE MC, KRAILO MD, HENDERSON BE, DUKE A AND ROY A. (1983). 'Hormonal' risk factors, 'breast tissue age' and the age-incidence of breast cancer. *Nature*, **303,** 767 – 770.

REMENNICK LI. (1990). Induced abortion as cancer risk factor: a review of epidemiological evidence. *J. Epidemiol. Community Health*, **44,** 259 – 264.

ROSENBERG L. (1994). Induced abortion and breast cancer: More scientific data are needed. *J. Natl Cancer Inst.*, **86,** 1569 – 1570.

ROSERO-BIXBY L, OBERLE MW AND LEE NC. (1987). Reproductive history and breast cancer in a population of high fertility, Costa Rica, 1984 – 85. *Int. J. Cancer*, **40,** 747 – 754.

SOINI I. (1977). Risk factors of breast cancer in Finland. *Int. J. Epidemiol.*, **6,** 365 – 373.

TALAMIMI R, LA VECCHIA C AND FRANCESCHI S. (1985). Reproductive and hormonal factors and breast cancer in a Northern Italian population. *Int. J. Epidemiol.*, **14,** 70 – 74.

TAO SC, YU MC, ROSS RK AND KUANG WX. (1988). Risk factors for breast cancer in Chinese women of Beijing. *Int. J. Cancer*, **42,** 495 – 498.

TULINIUS H, SIGVALDASON H, HRAFNKELSSON J, OLAFSDOTTIR G, TRYGGVADOTTIR L AND SIGUROSSON K. (1990). Reproductive factors and breast cancer risk in Iceland. *Int. J. Cancer*, **46,** 972 – 975.

URSIN G, ZIEGLER RG, PIKE MC, WU AH, HOOVER RN AND WEST DW. (1995). Oral contraceptive use and breast cancer risk among Asian-American women. *Am. J. Epidemiol.*, **141,** S52.

WANG QS, ROSS RK, YU MC, NIN JP, HENDERSON BE AND KIMM HT. (1992). A case – control study of breast cancer in Tianjin, China. *Cancer Epidemiol Biomarkers Prev.*, **1,** 435 – 439.

WILLIAMS EMI, JONES L, VESSEY MP AND McPHERSON K. (1990). Short-term increase in risk of breast cancer associated with full term pregnancy. *Br. Med. J.*, **300,** 578 – 579.

YOO K-Y, TAJIMA K, KUROISHI T, HIROSE K, YOSHIDA M, MIURA S AND MURAI H. (1992). Independent protective effect of lactation against breast cancer: a case – control study in Japan. *Am. J. Epidemiol.*, **135,** 726 – 733.

YUAN JM, YU MC, ROSS RK, GAO YT AND HENDERSON BE. (1988). Risk factors for breast cancer in Chinese women in Shanghai. *Cancer Res.*, **48,** 1949 – 1953.

ZIEGLER RG, HOOVER RN, PIKE MC, HILDESHEIM A, NOMURA AMY, WEST DW, WU-WILLIAMS AH, KOLONEL LN, HORN-ROSS PL, ROSENTHAL JF AND HYER MB. (1993). Migration patterns and breast cancer risk. *J. Natl Cancer Inst.*, **85,** 1819 – 1827.